# Author’s reply to “Second atrial septum, or interatrial fold?”

**DOI:** 10.1016/j.hroo.2026.03.005

**Published:** 2026-03-10

**Authors:** Chizute Ogbedeh

**Affiliations:** University of Cambridge School of Clinical Medicine, Cambridge, United Kingdom

We thank Professor Anderson for his interest in our case report describing a complication of atrial fibrillation ablation^1^ and for his insightful remarks regarding the anatomy of the oval fossa and its surrounding structures.^2^

We described the traumatized area as an “interatrial septum” merely to reflect common electrophysiological terminology; nonetheless, we agree that precise anatomical nomenclature is important when discussing procedural complications. A thorough understanding of anatomy should accompany every operator performing interventional procedures, as it is often underestimated or oversimplified—as occurred in our case. Moreover, it reinforces our take-home message to avoid positioning the transseptal puncture catheter in close proximity to this vulnerable fold.

We hope that this clarification will aid future decisions for our colleagues regarding safe transseptal puncture.

## References

[1] Ogbedeh C., Monaco C., Ascione C., Jaïs P., Derval N., Hocini M. (2026). Inter-atrial septum dissection during atrial fibrillation ablation via atrial septal defect: a rare complication. Heart Rhythm O2.

[2] Anderson R.H., Brown N.A., Webb S. (2002). Development and structure of the atrial septum. Heart.

